# Unmet Needs of Primary Care Patients in Using the Internet for Health-related Activities

**DOI:** 10.2196/jmir.4.3.e19

**Published:** 2002-12-31

**Authors:** Christopher N Sciamanna, Melissa A Clark, Thomas K Houston, Joseph A Diaz

**Affiliations:** ^1^Brown Medical SchoolUSA; ^2^University of Alabama-Birmingham School of MedicineUSA

**Keywords:** Internet, primary care, patients, access to information

## Abstract

**Background:**

Millions of people use the Internet as a source for health information yet little is understood about the use of the Internet for other health-related activities.

**Objective:**

We conducted the present study to understand, among primary care patients, the interest in and experience with using the Internet for a variety of health-related activities.

**Methods:**

Cross-sectional survey in the setting of 4 community-based primary care practices in Rhode Island. A single self-administered questionnaire included the following: 14 items measuring interest in using the Internet for a variety of health-related purposes, demographics, self-reported health status, and self-reported health care quality.

**Results:**

The survey was completed by 300 patients, 109 without access to the Internet and 191 with access to the Internet. Experiences with and attitudes about each of the health-related activities on the Internet varied widely across each activity. Regardless of access, patients were most interested in using the Internet for finding information about diseases and medications. However, patients with Internet access were more interested, compared to those without access, in each of the health-related activities on the Internet. Among patients with access to the Internet, the largest gap between interest and experience (the *opportunity gap*) was in using the Internet to investigate the quality of their care (eg, "find out if your health care provider was giving you all of the tests and treatments that you are due to have?") and administrative functions (eg, "schedule an appointment with your doctor?").

**Conclusions:**

Much opportunity remains for developing health-related Internet Web sites to address the unmet needs of primary care patients.

## Introduction

The Internet continues to evolve as an increasingly-important source of health information for millions. Based on a national survey in March 2002, an estimated 73 million Americans have used the Internet for health information, with approximately 6 million Americans going online for health advice each day [1,2]. With an estimated 100000 health related Web sites, the Internet has changed the way that Americans access health information [3]. Patients use the Internet to investigate many health-related topics commonly encountered by primary care providers [4]. It is quite likely that 1 hour of Internet searching by an intelligent patient on a reputable Web site can give the patient information about his or her condition that the physician is not aware of [5]. Previous reports indicate that patients feel that information on the Internet is "better than" information from their doctor [4]. In fact, patients with lower self-rated health (ie, sicker patients) are the most likely to talk to health care providers about the information they found on the Internet [6]. Thus, this revolution in health care information has great potential to affect the way that patients interact with their physicians.

Furthermore, the pace of eHealth development has meant that more and more traditionally offline health-related activities can now be done online. For example, in addition to researching their medical conditions and seeking second opinions, patients can now access information about the quality of care in hospitals (http://www.healthgrades.com/ [7], order prescription drugs online (http://www.drugstore.com/),schedule an appointment with their doctor, participate in thousands of medical discussion groups (http://groups.yahoo.com/), and seekmedical advice from experts in various fields [8]. A select few patients have begun to use e-mail to communicate with their physician [9] and surveys suggest that many more desire the convenience of electronic mail with their provider [10].

This revolution in health care information has great potential to affect the way that patients interact with their physicians. Though many studies have examined the information available on the Internet, both in terms of patient's experiences and the quality of the information, little work has been done to evaluate the use of the Internet for other health-related activities, such as finding information about the quality of care that a hospital provides (http://www.leapfroggroup.org/hospital.htm) [3,11-15]. What eHealth activities do primary care patients most desire? This question has great relevance to health care providers as it is likely that patients use the Internet, at least in part, to make up for deficiencies in the health care system. Most notable among these deficiencies are physicians' lack of adherence to guidelines [16,17], the chasm between care that is received and that which is possible [18], and the 31% of 6722 adults in a recent national survey by the Commonwealth Fund who did not have a great deal of confidence in their doctor [18].

Although access to many Internet-based health care activities is still limited, the potential for patient-centered applications is broad. We conducted the present study to understand, among primary care patients, the interest in and experience with using the Internet for a variety of health-related activities including electronic communication with health care providers, use of electronic records, ordering medications, and assessing the quality of care. We have defined the difference between what people are doing and what people are interested in doing on the Internet as the *opportunity gap*. Identifying this opportunity gap is important for future development of Internet health-related activities, as consumer demand is felt to be one of the most influential drivers of Internet health-related activities (eHealth) over the next 5 years [19].

## Methods

We recruited a convenience sample of 4 community-based primary care practices from Providence County, Rhode Island. Physicians in each practice were affiliated with the Brown University teaching-hospital network. One of the practices was a state-supported, suburban public-health clinic serving low-income individuals, while the other 3 were suburban, primary care practices. The practices had an average of 3 full-time physicians on staff. The demographic profile for Providence County includes: 14.6% over the age of 65, 13.4% Hispanic or Latino, 6.5% Black or African American, and 15.5% of people living below the federal poverty level [20].

A research assistant approached 355 consecutive adult outpatients from June 1, 2001 to August 15, 2001, to complete a self-administered questionnaire. Subjects were paid $20 to complete the survey. The Institutional Review Board of The Miriam Hospital approved the protocol.

### Measures

In order to identify online health-related activities, 20 subjects were recruited for 2 focus groups by e-mailing notices to employees of The Miriam Hospital and Rhode Island Hospital and placing posters in public areas in both hospitals. Focus group participants were asked to identify health-related activities that they currently performed on the Internet. Health-related activities that were noted more than once or that the investigators felt were emerging Internet capabilities were added to the Internet Interest and Experience Survey below.

### Internet Interest and Experience Survey (IIES)

Questionnaire items were created to measure the interest of subjects in using the Internet for each of 14 potential activities, such as to "find information about a specific disease or medical condition" and to "find out what questions you should ask your doctors when you see them?" All 14 items were asked of each subject, regardless of whether or not they currently had access to the Internet. The 14 items are in the Appendix.

### Sample Descriptors

Brief screening questions for age, gender, educational attainment, health insurance status, perceived health, and race and ethnicity were adapted from the year-2000 Behavioral Risk Factor Surveillance System (BRFSS) [21]. Internet use was assessed using questions adapted from the Pew Internet and American Life Project [2]. A single item was used to measure perceived quality of care. The item was used previously in the 2000 Behavioral Risk Factor Surveillance System [21]. The item asked patients to rate the quality of "all your health care" on a scale from 1 to 5 where 1 is the "worst health care possible" and 5 is the "best health care possible."

### Data Analysis

Many of the Internet Interest and Experience Survey (IIES) items had a bimodal distribution, so IIES items were categorized into *more interested*(4, 5 = 1) and *less interested*(1, 2, 3 = 0). First, we compared patients' interest in using the Internet for health-related activities by whether or not they had access to the Internet. Second, among patients with access to the Internet, we subtracted the percentage of patients who had used the Internet for each health-related activity from the percentage who expressed an interest in using the Internet for each health-related activity. We defined this difference in percentage as the *opportunity gap*, the percentage of patients whose needs for using the Internet for health-related activities may not be being met.

All data analyses were carried out using SPSS for Windows, version 10.0.5. Chi-square tests and analysis of variance were used to examine differences in categorical data among variables with 2 categories (eg, age and gender), and more than 2 categories (education level only), respectively. Due to the presence of some missing data, some analyses include fewer than 300 individuals. No variable included in our analysis was missing for more than 2% of the sample.

## Results

The survey was completed by 300 patients, for a response rate of 84.5%. Approximately two-thirds (63.7%) reported having Internet access at home, work, school, family or friend's home, or at a library. The mean patient age was 45.2 years (range: 18-75 years), 83.0% (n = 249) were female, 21.3% (n = 64) had completed at least 4 years of college, and 9.7% (n = 29) had no health insurance.

Table 1 shows the bivariate associations between Internet access and background characteristics. Internet access was greater among subjects who were younger, who had more formal education, and who had better self-reported health. Internet access was not related to gender, race, health insurance status, or perceived quality of care.

**Table 1:** Background characteristics by Internet access

	**Without Access (n = 109)%**	**With Access (n = 191)%**	**Pearson χ^2^**
Age 18-54 > 54	21.1 71.4	78.9 28.6	69.5, df = 1 P = .000
Gender Male Female	33.3 36.9	66.7 63.1	.2, df = 1 P =.63
Education Less than high school High school/some college completed College graduate	65.6 38.2 15.6	34.4 61.8 84.4	24.0, df = 2 P = .000
Race White Non-white	37.6 31.2	62.4 68.8	.7, df = 1 P = .40
Health insurance status Insured Not insured	35.4 44.8	64.6 55.2	1.0, df = 1 P = .32
Self-reported health rating Excellent/very good Good/fair/poor	23.9 76.1	39.8 60.2	7.8, df = 1 P = .005
Perceived quality of care 5 (best care) 4/3/2/1 (less than best care)	47.7 52.3	36.6 63.4	3.5, df = 1 P = .06

Table 2 shows the self-reported interest in and experience of subjects with the use of the Internet for each specific health-related activity, limited to those with access to the Internet. Percentages in Table 2 reflect individuals who were more interested in using the Internet for each health-related activity; the health-related activities are listed in order of descending opportunity gap. Among all subjects, interest was greatest in using the Internet to: (1) find information about a disease (61.7%), (2) find information about a medication (55.4%), and (3) find out what questions they should be asking during doctor visits (48.0%) (data not shown). Patients with Internet access were more interested, compared to those without access, in all of the Internet health-related activities asked in the questionnaire (data not shown).

Subjects with access to the Internet most frequently reported using it to: (1) find information about a disease (67.0%), (2) find information about a medication (53.4%), and (3) to help modify their lifestyle (such as quitting smoking) (46.6%). The greatest opportunity gap existed for using the Internet to: (1) find out if their health care provider is giving them the tests and treatments they need (39.8%), (2) schedule an appointment with their doctor (36.6%), and (3) find out how the quality of care their doctor providers compares to other doctors (35.9%). For example, in Table 2, 57% of patients with Internet access expressed an interest in using the Internet to find out if their health care provider is giving them the tests and treatments they need, compared to only 17.3% who reported ever doing this on the Internet. The smallest opportunity gap (6.0%) existed for using the Internet to assist in lifestyle modifications, where 52.6% of subjects reported an interest in this online activity and 46.6% said they had already done this on the Internet.

**Table 2:** Self-reported interest and experience of primary care patients with the use of the Internet for specific health-related activities among patients with Internet access (n = 191) - percentages reflect individuals who were more interested in using the Internet for each health-related activity

Health-related activity*	**Interest in**† **Internet** **% of patients (number of individuals responding to each item)**	**Experience with**‡ **Internet** **% of patients (number of individuals responding to each item)**	**Opportunity gap**§ **(number of individuals reflected in opportunity gap percentages)**
Find out if your health care provider was giving you all of the tests or treatments that you were due to have?	57.1 (191)	17.3 (191)	39.8 (76)
Schedule an appointment with your doctor?	44.0 (191)	7.4 (191)	36.6 (70)
Find out how the quality of care your doctor provides compares to other doctors?	50.0 (191)	14.1 (190)	35.9 (68)
Find out what questions you should ask your doctors when you see them?	57.6 (191)	24.6 (191)	33.0 (63)
Find out what tests or treatments you are due to have when you see your doctor?	53.9 (191)	25.1 (191)	28.8 (55)
Create your own personal, online, medical chart of your past illnesses, tests and treatments?	28.4 (191)	5.8 (190)	22.6 (43)
Find information about the quality of care a hospital provides?	49.7 (191)	28.8 (191)	20.9 (40)
Find information about the quality of care a doctor provides?	47.6 (191)	33.0 (191)	14.6 (28)
Find a doctor to see for your personal care?	35.3 (191)	21.5 (191)	13.8 (26)
Buy medications from a pharmacy?	30.4 (191)	17.3 (191)	13.1 (25)
Send an email (electronic mail message) to a doctor, nurse, or other health professional?	32.3 (190)	19.4 (189)	12.9 (24)
Look for information about a medication?	65.8 (191)	53.4 (190)	12.4 (23)
Find information about a specific disease or medical condition?	74.9 (191)	67.0 (191)	7.9 (15)
Help you modify your lifestyle? (examples include quitting smoking, becoming physically active, losing weight, changing your diet)	52.6 (191)	46.6 (190)	6.0 (11)

^*^ Listed in order of descending opportunity gap.

*Interest*= responding 4 or 5 on a scale where 1 was "not at all interested" and 5 was "extremely interested" in using the Internet for the specific purpose.

*Experience*= responded "yes" that subject had used the Internet for the specific purpose.

^§^ Opportunity gap = percentage of patients having *interest in Internet*(†) minus percentage of patients having *experience with Internet*(‡).

## Discussion

The Internet is changing the doctor-patient relationship as it provides patients with the potential to make better health decisions via easy access to vast amounts of health information. In the present study, we attempted to investigate the interest in and experience with using the Internet for a variety of health-related activities among a group of primary care patients in Rhode Island. The main findings were that great gaps exist between the health-related activities patients are currently doing on the Internet and the activities they would like to be doing.

For 4 of the 14 Internet health-related activities in the questionnaire, there was an opportunity gap (see definition in Data Analysis section of Methods) of greater than 30%. Three of the 4 health-related activities with the largest opportunity gap were related either directly or indirectly to health care quality, including using the Internet to: (1) "find out if your health care provider was giving you all of the tests or treatments that you are due to have?", (2) find out how the quality of care your doctor provides compares to other doctors?", and (3) "find out what questions you should ask your doctors when you see them?" Another area of opportunity focused on the ability of the Internet to perform administrative functions, including appointment scheduling and creating an online chart. Increasing transparency in quality of care has become a major policy issue, as highlighted in the recent Institute of Medicine report, Crossing the Quality Chasm [18].

Based on our survey, the Internet is meeting the needs of primary care patients for information about diseases and medications, because for each of these activities the majority of patients were interested and the opportunity gap was small. With regards to e-mail, fewer patients in our survey with Internet access were interested in e-mailing their health care providers than noted by Sittig and colleagues (32% vs 65%, respectively) [11]. This difference is most likely due to response bias in the study by Sitttig and colleagues, which reported data on a survey e-mailed to registered users of http://www.webmd.com/, which had aresponse rate of only 15.9%.

Web sites designed to enable patients to learn about the quality of care they are receiving and the questions they should be asking during doctor visits currently do exist, but are relatively uncommon. Two sites in particular, http://www.doctorquality.com/ http://www.healthgrades.com/,allow patients to rate health care providers and facilities, but currently have limited functionality due to the lack of high-quality data upon which to base information. On the other hand, many Web sites have suggested questions that patients should ask their doctor (eg, http://www.niddk.nih.gov/health/diabetes/pubs/bldsgr/bldsgr.htmand http://www.americanheart.org/presenter.jhtml?identifier=4678).Patients may not know these sites or may not perceive them to be useful. Future studies will be necessary to understand how best to either (1) develop Internet resources to address these unmet needs or (2) make the Web sites that meet these needs more available or known to online health seekers.

Another area of potential development focused on the ability of the Internet to perform administrative functions, including appointment scheduling and creating an online chart. Although some patients may have used e-mail with their provider to perform administrative functions [9], use of this technology is not widespread and is not well integrated into the triage and traditional workflow of clinical care. New programming standards, including Extensible Markup Language (XML), that increase the flexibility of the Internet for use as a data warehouse may accelerate the development of these applications [22]. Electronic medication-refill requests integrated with physician decision support systems could potentially reduce errors and be cost-effective [23]. Thus, this Internet-based administration of health care may be desirable to both health care consumers and payers.

If the history of the Internet has been a teacher, then the findings in our survey have great implications for health care providers, insurers, and hospitals. Content on the Internet has generally tracked consumer demand because the two most common ways that Web sites are funded is either by advertising revenue or by subscriptions. Our study suggests that patients want to start to use the Internet not only as a source of information about conditions and medications, but also as a way to inform their health care decisions. Half of our respondents were interested in using the Internet to find out about the quality of care their doctor provides and several Web sites are currently being developed specifically for this purpose (eg, http://www.doctorquality.com/www/, http://www.leapfroggroup.org/).Should history prove correct, that the Internet evolves to deliver what consumers desire, our data suggest that consumers may have the greatest potential as a driver of improving health care quality.

The study, however, has several noteworthy limitations:

- First, our survey did not measure every current health-related activity available on the Internet, because we based questions on the responses of our focus group participants and on the knowledge of the investigators. For example, support groups and health risk appraisal sites (eg, http://www.realage.com/, http://www.yourcancerrisk.harvard.edu/, http://chess.chsra.wisc.edu/Chess/)are common on the Internet [24-28], but our survey did not measure the use or interest in these online health-related activities.
- Second, our measurement of the opportunity gap for online health-related activities was intuitive but somewhat arbitrary. For example, we did not measure the degree to which patients' perceived needs were met by each online health-related activity. Diaz and colleagues reported, however, that health information on the Internet was generally perceived as quite useful, ranked second only to health information from a physician or nurse [4]. Also, though the opportunity gap for lifestyle modification over the Internet was small, few sites that offer personally tailored information exist [29]. From a public health perspective, delivery of interactive, tailored health information can be effective in changing patient health behaviors [30,31]. Future studies should examine this issue in more detail.
- Third, though our response rate was greater than 80%, our survey was only done in 4 primary care practices in Rhode Island, therefore it may not generalize to other populations or settings.
- Fourth, our survey relied on self-report of Internet health-related activities. Future studies should consider methods such as installing software on patients' computers to record their Internet activities [3].

Despite the study's limitations, the results of this study have important implications as the number of patients using the Internet for health-related activities continues to grow. People frequently use the Internet to gather health information; about 6 million Americans do so each day [1]. This study confirms these findings but also identifies additional activities where patients show interest in furthering their use of the Internet. As the spectrum of available health-related Internet activities expands, patients may soon use the Internet to research the quality of physicians, schedule their own appointments, and investigate the quality of care they receive. The traditional doctor-patient relationship will continue to evolve as health care on the Internet advances.

Future studies are needed to address this rapidly-evolving technology. Given that the field is quite new, valid and reliable measurements need to be developed. The majority of the data on Internet use is collected using self-reported surveys, as in the present study, yet little is known regarding the validity or reliability of such survey data on Internet use [1]. Without these improved methodologic approaches, the science of the field will move slowly, as studies of doctor-patient communication have been hampered by over-reliance on survey data, rather than more-valid and more-reliable methods, such as videotape and audiotape methods [32]. Eysenbach and colleagues made an important step in this direction by videotaping sessions in which individuals were asked to find specific health-related information on the Internet [3]. Another approach is to incorporate technologies that track and record the sites visited and activities performed on health-related Web sites [3]. Given the proliferation of health-related activities on the Internet, these methodologic advances are greatly needed to advance research in the field.

## Abbreviations

IIES: Internet Interest and Experience Survey

## Appendix 1

(Each numbered question was asked in two ways.)

Please answer the following questions based on a scale of 1 to 5 where 1 is "not at all interested" and 5 is "extremely interested".

- How interested are you to use the Internet to...
- Have you ever personally used the Internet to...

1. Send an e-mail (electronic mail message) to a doctor, nurse, orother health professional?
2. Find information about the quality of care a doctorprovides?
3. Find information about the quality of care a hospitalprovides?
4. Find out what tests or treatments you are due to have when yousee your doctor?
5. Schedule an appointment with your doctor?
6. Help you modify your lifestyle? (examples include quittingsmoking, becoming physically active, losing weight, changing yourdiet)
7. Find a doctor to see for your personal care?
8. Find information about a specific disease or medicalcondition?
9. Find out if your health care provider was giving you all of thetests or treatments that you were due to have?
10. Find out how the quality of care your doctor provides comparesto other doctors?
11. Find out what questions you should ask your doctors when you seethem?
12. Buy medications from a pharmacy?
13. Look for information about a medication?
14. Create your own personal, online, medical chart of your pastillnesses, tests and treatments?
